# Contribution of Primary Pelvic Organ Prolapse to Micturition and Defecation Symptoms

**DOI:** 10.1155/2012/798035

**Published:** 2011-09-29

**Authors:** Annette G. Groenendijk, Erwin Birnie, Jan-Paul W. Roovers, Gouke J. Bonsel

**Affiliations:** ^1^Department of Gynecology and Obstetrics, Onze Lieve Vrouwe Hospital, P.O. Box 95500, 1090 HM Amsterdam, The Netherlands; ^2^Institute of Health Policy and Management, Erasmus Medical Center, P.O. Box 1738, 3000 DR Rotterdam, The Netherlands; ^3^Department of Gynecology and Obstetrics, Academic Medical Center, University of Amsterdam, P.O. Box 22660, 1100 DD Amsterdam, The Netherlands; ^4^Department of Obstetrics and Gynecology and Division Obstetrics and Prenatal Medicine, Erasmus MC-Sophia, P.O. Box 2060, 3000 CB Rotterdam, The Netherlands

## Abstract

*Objective*. To investigate the contribution of Pelvic Organ Prolapse (POP) to micturition and defecation symptoms. *Method*. Cross-sectional study including 64 women presenting with POP symptoms and 50 controls without POP complaints. Subjects were evaluated using POP-Quantification system, Urinary Distress Inventory, and Defecation Distress Inventory. The MOS SF-36 health survey and the Center for Epidemiological Studies Depression scale were used to measure self-perceived health status and depressive symptoms, respectively. *Results*. POP in terms of POP-Q had a moderate impact on the symptom observing vaginal protrusion (explained variance 0.31). It contributed modestly to obstructive voiding and overactive bladder symptoms (explained variance 0.09, resp., 0.14) but not to urinary incontinence. Constipation was more likely explained by clinical depression than by pelvic floor defects (explained variance 0.13, resp., 0.05). *Conclusion*. Stage of POP and specific prolapse symptoms are associated but such a strong association does not exist between POP and micturition or defecation symptoms.

## 1. Introduction

Pelvic organ prolapse (POP) is a common disorder often associated with symptoms such as a vaginal bulging, pelvic heaviness, bothersome micturition, and defecation symptoms as well as sexual dysfunction, often with a negative impact on quality of life [1, 2]. It is unclear whether the anatomical position of the bladder, bowel, and uterus compromises the bladder and bowel function directly, or whether abnormal anatomy and dysfunction of the pelvic floor share a common etiology. Moreover, it is unclear to what extent micturition and defecation symptoms can be explained by the presence and degree of anatomical abnormalities involved in POP. With the exception of vaginal bulging, none of these symptoms are specific to vaginal prolapse since they also exist in women without POP [3]. Whether or not the symptoms are related to POP is critical to patient management. POP patients in whom defecation symptoms dominate might be primarily referred to the gastroenterologist, but if these patients present with a vaginal prolapse, these patients are usually referred to the gynecologist. The latter commonly offers POP surgery with the correction of the anatomy as well as restoration of the pelvic floor function as treatment aims. This treatment policy assumes a causal rather than indirect relation between POP and these symptoms. However, surgical results frequently are disappointing in terms of pelvic floor function and symptoms [4, 5]. 

In this study we address the unclear relation between POP and pelvic floor symptoms and compare women who present with symptomatic POP with asymptomatic women. We investigated to what extent bladder and bowel symptoms are related to specific anatomical defects of the pelvic floor or to other factors like patient characteristics (e.g., age, parity, body weight, educational level) and psychological characteristics.

## 2. Material and Methods

We conducted a cross-sectional study between January 2000 and January 2002 consisting of two groups. The study group consisted of 64 women with symptomatic POP stage 2 or more treated at the gynecology outpatient clinic of the Onze Lieve Vrouwe Hospital. These patients participated in a larger study on the evaluation of the diagnostic work-up of patients with symptomatic primary POP [6–8]. The control group consisted of 35 women who were referred to the gynecology outpatient clinic for other complaints but not seeking medical care for POP and 15 women without gynecological complaints and who were not referred. 

Exclusion criteria for both groups were being less than 6 months postpartum, having congenital defects of the urogenital and/or gastrointestinal tract, a fibroid uterus with a size of more than 12 weeks of pregnancy, large ovarian cysts, prolapse surgery and/or hysterectomy in medical history, a poor general condition precluding surgical therapy or insufficient Dutch language proficiency. Patients who visited the general gynecologic outpatient clinic were excluded from the control group if they appeared to have symptoms of pelvic prolapse.

The study was approved by the Medical Ethical Board of the Onze Lieve Vrouwe Hospital. 

Standardized medical review and physical examination were carried out during the first visit at the gynecology outpatient clinic of the Onze Lieve Vrouwe Hospital. Stage of POP was assessed using the POP-Quantification (POP-Q) system with the patient sitting 45 degrees upright in a gynecological examination chair while she was instructed to strain forcefully [9]. In agreement with the study of Kahn and colleagues, we used the sum of the anatomic landmarks genital hiatus (gh) and perineal body (pb) as measure for perineal descent [10]. All pelvic examinations were performed by the first author (A. G. Groenendijk). In addition to a standard history review, each patient was invited to complete the following surveys. (1) The MOS SF-36 generic health-related quality-of-life questionnaire was used to measure self-perceived health status [11]. We used the overall physical and mental health summary scores (score range: 0–100, a higher score indicates better health) as indicators of physical and mental health. (2) The Center for Epidemiological Studies Depression scale (CES-D) was used to measure depressive symptoms [12] (scores range: 0 (no symptoms)−60(maximal symptoms); a cutoff score of 16 indicates clinical depression). (3) For the measurement of urogenital and bowel symptoms and symptom-related bother, we used two disease-specific symptom questionnaires. Firstly, the 19-item urinary distress inventory (UDI) consists of five domains: genital prolapse (e.g., feeling and/or seeing a vaginal bulge), urinary incontinence (e.g., urine leakage related to physical activity, coughing, or sneezing and urine leakage related to the feeling of urgency), overactive bladder (e.g., frequency, urgency, and nocturia), obstructive micturition (e.g., feeling of incomplete bladder emptying and difficulties to empty the bladder), and discomfort/pain (e.g., lower abdominal pressure, pain or discomfort lower abdomen, push on the vaginal wall to have bowel movement). Secondly, we used the 15-item defecation distress inventory (DDI) consisting of four domains: constipation, fecal incontinence, painful defecation, and incontinence for gas. The constipation domain was covered by the following items: less than 3 bowel movements a week, in 25% of the time straining at defecation, feeling of incomplete evacuation, sensation of anal blockage, and difficulties with emptying the rectum (manual removal of feces out of the rectum or push on the vaginal wall). Each domain score ranges from 0 to 100, and a higher score indicates more bother of reported symptoms [13, 14]. Both questionnaires have been validated in the Dutch language.

## 3. Analysis

Differences in stage of pelvic organ prolapse, patient characteristics, and reported pelvic floor symptoms between the study and the control group were evaluated using the Student's independent samples *t*-test for Gaussian distributed variables, the nonparametric Mann-Whitney *U*-test for skewed variables, and the chi-square test for nominal/ordinal variables. 

The impact of (1) individual risk factors (age, body mass index (BMI), parity, perineal trauma, summary physical health (as proxy for comorbidity), and educational level), alongside (2) specific pelvic floor defects (anterior, middle, and posterior compartment defects) and (3) psychological health status (clinical depression and summary mental health) on the UDI and DDI domain scores (both log transformed) was assessed using multiple linear regression analysis. The resulting beta-coefficients represent the impact on the log UDI or log DDI domain score when the risk factor is changed with one unit of measurement. Adjusted *R* ^2^ was used as measure of model fit. The change in adjusted *R* ^2^ was used to quantify the contribution of patient characteristics, psychological health, and specific pelvic floor defects, respectively (in this order, stepwise multiple linear regression analysis).

The post hoc sample size estimation showed that at least 84 patients had to be included in the analysis (power 80%) or alternatively 111 patients (power 90%) (type I error (alpha) = 0.05 (two sided), 13 predictors (see Table 2), effect size = 0.25 corresponding to *R* ^2^ = 0.20). 

SPPS for Windows version 16.0 was used for data management and statistical analysis. A two-sided *P* value <0.05 was considered a statistically significant difference.

## 4. Results

Table 1 depicts the characteristics of the 64 women of the study and the 50 women of the control group. Women in the study group were on average older and had higher parity as compared to the control group. Patients in the study group on average had higher POP stage compared to the control group (POP III/IV: 74% versus 4%, resp.), and their physical health was significantly worse. 

The study group reported significantly more bother from urogenital and bowel symptoms. Significant differences between the groups were found for all UDI domains as well as the flatus and fecal incontinence domains of the DDI. Feeling of vaginal protrusion (50/64 (78%)) and overactive bladder symptoms (45/64 (70%)) were the most frequent and bothersome symptoms in the study group followed by complaints of discomfort and pain. In the control group, the most prevalent and bothersome complaint was urinary incontinence (22/50, 44%). Frequently reported defecation symptoms in both groups were false urge for defecation (27/64 (42%) and 17/50 (34%), resp.), obstructed defecation (29/64 (45%) and 12/50 (24%)), and feeling of incomplete defection (29/64 (45%) and 8/50 (16%), resp.). 

Table 2 shows the association between micturition (UDI) and defecation (DDI) scores on the one hand and patient's characteristics, POP-Q scores, and psychological characteristics on the other.

### 4.1. Prolapse Feeling

Pelvic floor defects, dominated by anterior vaginal wall prolapse (Ba) and perineal descent of the pelvic floor (gh + pb), accounted for 31% (*P* < 0.001) of the variance in symptom scores. The impact of clinical depression and summary mental health on prolapse feeling was small (variance explained: 2%, *P* = 0.14). Patient characteristics overall explained 14%. A higher BMI adjusted for other covariables was associated with lower scores of prolapse feeling. 

### 4.2. Obstructive Voiding

Voiding problems were associated to specific pelvic floor defects but the overall contribution was modest (9% of explained variance, *P* = 0.004). Voiding problems were predominantly related to patient characteristics, explaining 21% of variance (*P* < 0.001). The relationship between presence of perineal trauma and voiding obstruction was inverse. Patients with better overall physical health had significantly less bother from voiding obstruction, but the impact was small.

### 4.3. Overactive Bladder

Overactive bladder symptoms were mainly related to pelvic floor defects (14% of variance explained, *P* = 0.001), especially to posterior vaginal wall prolapse (point Bp) and to a lesser extent point C. The contribution of patient characteristics was small (7% of variance explained). Only educational level had a significant impact on overactive bladder symptoms, that is, women with lower vocational education had more bother of overactive bladder symptoms.

### 4.4. Discomfort and Pain

Discomfort and pain were mainly related to pelvic floor defects (12% of variance explained), especially posterior vaginal wall prolapse (Bp) and perineal descent of the pelvic floor (gh + pb). However, discomfort and pain scores were also partially explained by patient and psychological characteristics; patients with lower overall physical health and clinically depressed patient showed more discomfort and pain.

### 4.5. Constipation

Constipation was predominantly related to psychological factors (13% of variance explained). Particularly clinically depressed patients reported higher levels of constipation. Constipation was to a lesser extent also related to pelvic floor defects (5% of variance explained, *P* = 0.033); particularly perineal descent (gh + pb) had a significant impact on constipation (*P* = 0.03). Of the patient characteristics only BMI and physical health were significantly related to constipation.

### 4.6. Other UDI and DDI Domains

None of the covariables studied had a significant impact on urinary incontinence (UDI) and fecal incontinence, painful defecation, and incontinence for gas (DDI).

We also examined the association of mild or more severe prolapse with urinary incontinence. In the mild prolapse group (overall POP-Q stages I and II; *n* = 65), the impact of anterior wall prolapse (represented by point Ba) on the UDI domain score (log transformed) with the same predictors as in Table 2 was beta = 2.75, 95%-CI: 0.04 to 1.25 (*P* < 0.01). In the severe prolapse group (overall POP-Q stages III and IV; *n* = 49) we found an inverse but not significant impact of anterior wall prolapse on urinary incontinence: beta= −0.18, 95%-CI: −0.51 to 0.16 (*P* = 0.28). 

## 5. Discussion

In this study the association between anatomical and functional abnormalities of the pelvic floor was poor. Anatomical defects and, to a lesser extent, patient characteristics were associated with obstructive voiding and overactive bladder but not with urinary incontinence. Any direct association between psychological factors and micturition symptoms appeared absent. Defecation symptoms were unrelated to anatomical abnormalities, patient characteristics, or psychological factors, except for constipation which was associated with psychological factors and, to a lesser extent, with perineal descent. Since the explanatory power of all pelvic floor symptoms was small, it is still unclear which are the main factors that underlie micturition and defecation symptoms.

Some limitations of this study need to be discussed. Firstly, although in agreement with other studies, only few of our patients presented with severe posterior compartment prolapse. As our study group represents an average distribution of vaginal prolapse patients, we do not believe this to be an important drawback. An overrepresentation of patients with severe posterior wall defects is likely to strengthen the relationship between posterior defects and defecation symptoms. Furthermore, forty percent of the women in the control group had a prolapse stage II according to the POP-Q classification system. This high prevalence of mild prolapse is in agreement with epidemiological studies that showed that up to 40% of women over the age of fifty years have mild asymptomatic prolapse [15, 16], which we regard as still a physiologic condition. Secondly, we did not document whether patients or controls had comorbidity. Instead, we used the SF-36 summary physical health dimension as a proxy measure. Probably, this is a more valuable measure to investigate whether pelvic floor function is associated with patient's general health status. Thirdly, since patients and controls had different characteristics, we adjusted for the documented prognostic factors in the multiple regression analysis. We do not think that prognostic incomparability plays an important role as all theoretical prognostic factors were documented in both groups.

Finally, there are two statistical limitations. We did not adjust the type I error level for multiple testing. Furthermore, regression analyses with multiple variables may have introduced multicollinearity or confounding. Multicollinearity did not occur as all bivariate correlations between covariables were <0.80. Removal of the POP-Q points from the regression model showed significant associations between parity and prolapse feeling (beta = 0.4, *P* = 0.003) and between age and fecal incontinence (beta = 0.04, *P* = 0.01). Although associations between covariables might affect the significance of the beta coefficients, they generally do not affect the *R* ^2^ of the model.

Although POP and urinary incontinence frequently coincided, we found no significant relationship between prolapse and overall urinary incontinence symptoms. An explanation could be that mild prolapse is associated with urinary stress incontinence but severe prolapse is more associated with continence and voiding dysfunction. This theory is supported by our findings from the stratified analysis, showing that mild anterior wall prolapse was found to be significantly associated with urinary incontinence but severe anterior wall prolapse was not. 

Furthermore, posterior compartment prolapse was associated with overactive bladder symptoms whereas, in contrast to what one may expect, anterior wall compartment prolapse was not. Only few studies on the relationship between POP and these symptoms are available. Some researchers found a relationship between anterior wall prolapse and overactive bladder symptoms due to outlet obstruction of the bladder [17], while others could not corroborate that association [18]. Our findings are in agreement with the findings of other reports that show that the site of POP and the type of pelvic floor symptoms are not consistently related [19, 20].

Experienced discomfort and pain in the pelvic area appeared to be related to clinical depression but from this study we cannot conclude whether this is a causal relationship or not. Furthermore we found that discomfort and pain symptoms were strongly related to posterior vaginal wall prolaps and perineal descent than to anterior vaginal wall prolapse. Maybe the feeling of pressure on the pelvic floor is caused by invisible structural abnormalities of the posterior compartment like enterocele [21, 22]. 

Surprisingly, we found no significant effect of age and parity on urogenital and defecation symptoms. One reason may be that the variation of these factors in our population was small, hampering the detection of significant associations. Alternatively, age and parity may have been undetected due to their associations with the respective POPQ points.

Furthermore, we found that a higher BMI was inversely related to prolapse feeling. While the literature supports overweight as a risk factor for pelvic organ prolapse [23], other studies show a protective effect of higher BMI level on pelvic floor injury [24, 25].

Defecation symptoms are frequently reported by women with POP [26] but whether they are the cause or the result of POP is unclear. Researchers report conflicting results about the relationship between the severity of prolapse and bowel symptoms [27, 28]. The association between pelvic floor defects and defecation symptoms in our study appeared to be small to absent. One explanation is that other factors than POP are predominantly responsible for defecation symptoms. The multifactorial pathophysiology of defecation disorders is likely to reduce the contribution of POP, that is, posterior vaginal wall prolapse amongst other factors, for example, occult anorectal anomalies, pelvic floor dyssynergia, endocrine and metabolic factors, and use of medication. The DDI we used to assess the presence of constipation is not valid to determine the symptom's etiology. While outlet obstruction seems responsible for the association between perineal descent and constipation, the association between clinical depression and constipation points to slow transit constipation as the result of different life style.

Another explanation could be that small and mild posterior wall prolapses as frequently found in our study group should be regarded a physiologic condition often present in women without defecation complaints [29] but too small to cause outlet obstruction. Finally, one may question whether POP-Q scores are the best representation of abnormalities of the rectovaginal wall [7, 30] since imaging techniques (defecography, MRI) can reveal anatomical abnormalities of the posterior compartment that are not represented by abnormal POP-Q scores [31]. 

Although POP and prolapse symptoms are associated, in our study we did not find a strong relationship between the affected compartment and most of the micturition and defecation symptoms. 

An explanation would be that prolapse and pelvic floor symptoms share a common aetiology rather than they have a direct causal relationship. Pathophysiologic concepts that might relate to prolapse and pelvic floor symptoms are collagen disease, abnormally weak pelvic floor muscles due to childbirth and pelvic floor neuropathy [32, 33]. The same neuropathy can obviously cause prolapse and a full range of bladder and bowel symptoms.

The above findings may have important clinical implications. In patients with mild POP who are not bothered by prolapse symptoms, surgical repair as treatment for functional disorders seems ill founded. In such cases, we first recommend conservative management of pelvic floor symptoms. Second, patients scheduled for POP surgery should be informed that coexisting micturition and defecation symptoms are not necessarily the result of POP and these may persist after surgery. The low proportion of explained variation in micturition and defecation symptoms stress the urge to further explore which factors determine the high prevalence of micturition and defecation symptoms in patients who present with POP. Improved insight into these factors may help to optimize the diagnostic work-up and treatment setting in patients with pelvic floor dysfunction.

## Figures and Tables

**Table 1:** Patient's characteristics.

Characteristics	Study group (*n* = 64)	Control group (*n* = 50)	*P *value
*Age (years), *mean (SD) [range]	56.1 (10.4) [35–77]	48.9 (8.1) [37–72]	<0.01
*Body Mass Index (BMI), *mean (SD) [range]^a^	25.38 (4.3) [18.4–39.1]	24.0 (4.3) [18.4–41.3]	0.99
Educational level			0.68
Primary school/lower vocational education	22 (34%)	6 (12%)	
Secondary school/high school	21 (33%)	23 (46%)	
Academic/university	22 (34%)	21 (42%)	
*Parity*			0.04
0	0 (—)	4 (8%)	
1	15 (23%)	21 (42%)	
2	22 (35%)	16 (32%)	
3	15 (23%)	8 (16%)	
≥4	12 (19%)	1 (2%)	
Overall, mean	2.3	1.6	
Type of delivery			0.36
Vaginal delivery, no forceps or vacuum	56 (88%)	40 (80%)	
Forceps and/or vacuum	8 (12%)	9 (18%)	
Caesarian section only	0 (—)	1 (2%)	
Perineal trauma (per patient)			0.52
No perineal trauma	16 (25%)	16 (32%)	
Episiotomy or rupture	48 (75%)	34 (68%)	
POP-Q points, mean (SD)^b^			
Aa	−0.8 (1.1)	−2.0 (1.2)	
Ba	2.2 (2.2)	−1.9 (1.3)	
C	−0.2 (4.1)	−5.7 (1.4)	
Gh	4.1 (1.1)	2.2 (0.7)	
Pb	2.7 (0.7)	2.9 (0.6)	
Ap	0.2 (1.4)	−1.6 (1.5)	
Bp	0.3 (1.6)	−1.5 (1.5)	
TVL	8.1 (1.1)	8.6 (1.1)	
POP-Q stage			<0.01
Stage I	0 (—)	24 (48%)	
Stage II	17 (27%)	24 (48%)	
Stage III	42 (66%)	2 (4%)	
Stage IV	5 (8%)	0 (—)	
UDI domains, median (IQR)			
Prolapse symptoms	33.3 (45.8)	0.0 (0.0)	<0.01
Obstructed voiding	16.7 (33.3)	0.0 (0.0)	<0.01
Overactive bladder	27.8 (30.6)	0.0 (22.2)	<0.01
Discomfort and pain	22.2 (31.9)	5.6 (16.7)	<0.01
Urinary incontinence	13.3 (26.7)	6.6 (13.3)	<0.01
UDI total	127.8 (69.1)	22.2 (35.0)	<0.01
DDI domains, median (IQR)			
Constipation	4.7 (17.3)	0.0 (9.5)	0.08
Fecal incontinence	0.0 (13.3)	0.0 (0.0)	0.02
Painful defecation	0.0 (0.0)	0.0 (0.0)	0.64
Flatus incontinence	16.7 (41.7)	0.0 (33.3)	0.02
DDI total	34.5 (67.4)	21.0 (45.4)	0.03
General health (MOS SF36), mean (SD)			
Summary mental health	46.3 (14.6)	50.3 (15.0)	0.42
Summary physical health	48.5 (11.3)	54.3 (11.7)	<0.01
Depression (CES-D)			
Clinical depression^c^	23 (36%)	13 (26%)	0.52

BMI: body mass index; IQR: interquartile range; SD: standard deviation.

^a^BMI of women in the normal population is 18–24 (body mass index = kg/m^2^).

^b^Point D was not correctly measured in all case and was excluded from the study.

^c^Defined as a CES-D score ≥16.

**Table 2:** Multiple linear regression analysis showing the relationship between patient's characteristics, psychological characteristics and anatomical defects at the one hand and pelvic floor symptoms (log scale) at the other hand.

	*β*-coefficient [95%-CI]	*P* value	Change in *R* ^2^ adjusted (*P* value of change)
(i) UDI-prolapse feeling (log scale)			

*Constant*	3.28 [−7.17 to 7.29]	0.11	
*Patient characteristics*			0.14 (*P* < 0.01)
Age (years)	0.00 [−0.03 to 0.04]	0.80	
BMI	−**1.00 **[−**0.17 to** −**0.3**]	<**0.01**	
Parity	0.14 [−0.08 to 0.36]	0.22	
Perineal trauma	−0.19 [−0.80 to 0.42]	0.54	
Physical health	−0.02 [−1.13 to 0.25]	0.28	
Educational level 1^a^	−0.20 [−0.73 to 0.72]	0.96	
Educational level 2^b^	−0.44 [−1.13 to 0.25]	0.21	
*Psychological factors*			0.02 (*P* = 0.14)
Clinical depression	0.65 [−0.02 to 1.32]	0.06	
Mental health	−0.01 [−0.04 to 0.02]	0.7	
*POP-Q points*			0.31 (*P* < 0.01)
Ba	**0.22 **[**0.04 ** **to 0.39**]	0.01	
C	0.04 [−0.07 to 0.15]	0.51	
Bp	0.17 [−0.02 to 0.36]	0.07	
gh + pb	**0.31 **[**0.05 to 0.58**]	**0.02**	
*Full model*			0.47 (*P* < 0.01)

(ii) UDI-obstructive voiding (log scale)			

*Constant*	4.69 [0.74 to 8.64]	0.02	
*Patient characteristics*			0.21 (*P* < 0.01)
Age (years)	−0.01 [−0.05 to 0.02]	0.44	
BMI	0.02 [−0.05 to 0.09]	0.55	
Parity	0.16 [−0.07 to 0.38]	0.17	
Perineal trauma	−**0.95 **[−**1.56 to **−**0.35**]	<**0.01**	
Physical health	−**0.06 **[−**0.09 to** −**0.02**]	<**0.01**	
Educational level 1^a^	0.44 [−0.30 to 1.17]	0.24	
Educational level 2^b^	−0.22 [−0.91 to 0.46]	0.52	
*Psychological factors*			0.00 (*P* = 0.35)
Clinical depression	−0.35 [−1.01 to 0.32]	0.30	
Mental health	−0.02 [−0.05 to 0.01]	0.24	
*POP-Q points*			0.09 (*P* < 0.01)
Ba	−0.03 [−0.20 to 0.14]	0.71	
C	0.10 [−0.00 to 0.21]	0.06	
Bp	0.09 [−0.09 to 0.28]	0.33	
gh + pb	0.18 [−0.09 to 0.44]	0.18	
*Full model*			0.30 (*P* < 0.01)

(iii) UDI-overactive bladder (log scale)			

*Constant*	3.09 [−0.91 to 7.09]	0.13	
*Patient characteristics*			0.07 (*P* = 0.04)
Age (years)	0.00 [−0.04 to 0.04]	0.99	
BMI	−0.01 [−0.08 to 0.06]	0.85	
Parity	−0.03 [−0.26 to 0.19]	0.77	
Perineal trauma	−0.33 [−0.94 to 0.28]	0.28	
Physical health	0.01 [−0.03 to 0.04]	0.67	
Educational level 1^a^	−**0.98 **[−**1.72 to** −**0.24**]	**0.01**	
Educational level 2^b^	−0.54 [−1.23 to 0.16]	0.13	
*Psychological factors*			0.00 (*P* = 0.49)
Clinical depression	0.27 [−0.40 to 0.94]	0.43	
Mental health	0.001 [−0.03 to 0.03]	0.97	
*POP-Q points*			0.14 (*P* < 0.01)
Ba	−0.02 [−0.15 to 0.19]	0.80	
C	0.11 [−0.00 to 0.21]	0.06	
Bp	**0.24 **[ **0.06 to 0.43**]	**0.01**	
gh + pb	−0.01 [−0.27 to 0.26]	0.97	
*Full model*			0.21 (*P* < 0.01)

(iv) UDI-discomfort and pain (log scale)			

*Constant*	4.93 [0.88 to 7.91]	0.01	
*Patient characteristics*			0.10 (*P* = 0.01)
Age (years)	−0.01 [0.05 to 0.02]	0.36	
BMI	−0.04 [−0.11 to 0.03]	0.25	
Parity	0.09 [−0.11 to 0.29]	0.36	
Perineal trauma	0.19 [−0.35 to 0.72]	0.49	
Physical health	−**0.04 **[−**0.07 to **−**0.01**]	**0.02**	
Educational level 1^a^	−0.09 [−0.74 to 0.57]	0.79	
Educational level 2^b^	−0.18 [−0.79 to 0.43]	0.56	
*Psychological factors*			0.07 (*P* < 0.01)
Clinical depression	**0.73 **[**0.14 to** **1.32**]	**0.02**	
Mental health	−0.01 [−0.04 to 0.01]	0.37	
*POP-Q points*			0.12 (*P* < 0.01)
Ba	−0.02 [−0.17 to 0.13]	0.79	
C	0.05 [−0.05 to 0.14]	0.35	
Bp	**0.17 **[**0.00 to 0.33**]	**0.05**	
gh + pb	**0.24 **[**0.00 to 0.47**]	0.05	
*Full model*			0.29 (*P* < 0.01)

(v) DDI-constipation (log scale)			

*Constant*	3.32 [−0.08 to 6.71]	0.06	
*Patient characteristics*			0.06 (*P* = 0.07)
Age (years)	0.00 [−0.03 to 0.03]	0.99	
BMI	−**0.08 **[−**0.14 to** −**0.02**]	**0.01**	
Parity	0.09 [−0.10 to 0.28]	0.35	
Perineal trauma	−0.12 [−0.64 to 0.40]	0.69	
Physical health	−**0.03 **[−**0.06 to **−**0.01**]	**0.02**	
Educational level 1^a^	0.58 [−0.31 to 0.87]	0.69	
Educational level 2^b^	0.28 [−0.31 to 0.87]	0.07	
*Psychological factors*			0.13 (*P* < 0.01)
Clinical depression	**0.96 **[**0.39 to** **1.53**]	<0.01	
Mental health	−0.01 [−0.04 to 0.01]	0.35	
*POP-Q points*			0.05 (*P* = 0.03)
Ba	−0.02 [−0.16 to 0.13]	0.82	
C	−0.08 [−0.17 to 0.02]	0.10	
Bp	0.12 [−0.04 to 0.28]	0.16	
gh + pb	**0.26 **[**0.03 to 0.49**]	**0.03**	
*Full model*			0.24 (*P* < 0.01)

^a^Secondary education; reference is primary school/lower vocational education.

^b^Higher professional education; reference is primary school/lower vocational education.

## References

[1] Digesu GA, Chaliha C, Salvatore S, Hutchings A, Khullar V (2005). The relationship of vaginal prolapse severity to symptoms and quality of life. *British Journal of Obstetrics and Gynaecology*.

[2] Jelovsek JE, Maher C, Barber MD (2007). Pelvic organ prolapse. *The Lancet*.

[3] Gutman RE, Ford DE, Quiroz LH, Shippey SH, Handa VL (2008). Is there a pelvic organ prolapse threshold that predicts pelvic floor symptoms?. *American Journal of Obstetrics and Gynecology*.

[4] Kahn MA, Stanton SL (1997). Posterior colporrhaphy: its effects on bowel and sexual function. *British Journal of Obstetrics and Gynaecology*.

[5] Milani R, Salvatore S, Soligo M, Pifarotti P, Meschia M, Cortese M (2005). Functional and anatomical outcome of anterior and posterior vaginal prolapse repair with prolene mesh. *British Journal of Obstetrics and Gynaecology*.

[6] Groenendijk AG, Birnie E, de Blok S (2009). Clinical-decision taking in primary pelvic organ prolapse; the effects of diagnostic tests on treatment selection in comparison with a consensus meeting. *International Urogynecology Journal and Pelvic Floor Dysfunction*.

[7] Groenendijk AG, van der Hulst VP, Birnie E, Bonsel GJ (2008). Correlation between posterior vaginal wall defects assessed by clinical examination and by defecography. *International Urogynecology Journal and Pelvic Floor Dysfunction*.

[8] Groenendijk AG, Birnie E, Boeckxstaens GE, Roovers JP, Bonsel GJ (2009). Anorectal function testing and anal endosonography in the diagnostic work-up of patients with primary pelvic organ prolapse. *Gynecologic and Obstetric Investigation*.

[9] Bump RC, Mattiasson A, Bo K (1996). The standardization of terminology of female pelvic organ prolapse and pelvic floor dysfunction. *American Journal of Obstetrics and Gynecology*.

[10] Kahn MA, Breitkopf CR, Valley MT (2005). Pelvic Organ Support Study (POSTT) and bowel symptoms: straining at stool is associated with perineal and anterior vaginal descent in general gynecologic population. *American Journal of Obstetrics and Gynecology*.

[11] Hays RD, Morales LS (2001). The RAND-36 measure of health-related quality of life. *Annals of Medicine*.

[12] Beekman ATF, Deeg DJH, Van Limbeek J, Braam AW, De Vries MZ, Van Tilburg W (1997). Criterion validity of the Center for Epidemiologic Studies Depression scale (CES-D): results from a community-based sample of older subjects in the Netherlands. *Psychological Medicine*.

[13] Van der Vaart CH, De Leeuw JRJ, Roovers JPWR, Heintz APM (2003). Measuring health-related quality of life in women with urogenital dysfunction: the urogenital distress inventory and incontinence impact questionnaire revisited. *Neurourology and Urodynamics*.

[14] van Brummen HJ, Bruinse HW, van de Pol G, Heintz APM, van der Vaart CH (2006). Defecatory symptoms during and after the first pregnancy: prevalences and associated factors. *International Urogynecology Journal and Pelvic Floor Dysfunction*.

[15] Samuelsson EC, Victor A, Tibblin G, Svardsudd KF (1999). Signs of genital prolapse in a Swedish population of women 20 to 59 years of age and possible related factors. *American Journal of Obstetrics and Gynecology*.

[16] Slieker-Ten Hove MCP, Pool-Goudzwaard AL, Eijkemans MJC, Steegers-Theunissen RPM, Burger CW, Vierhout ME (2009). Prediction model and prognostic index to estimate clinically relevant pelvic organ prolapse in a general female population. *International Urogynecology Journal and Pelvic Floor Dysfunction*.

[17] Basu M, Duckett J (2009). Effect of prolapse repair on voiding and the relationship to overactive bladder and detrusor overactivity. *International Urogynecology Journal and Pelvic Floor Dysfunction*.

[18] Schimpf MO, Sullivan DM, LaSala CA, Tulikangas PK (2007). Anterior vaginal wall prolapse and voiding dysfunction in urogynecology patients. *International Urogynecology Journal and Pelvic Floor Dysfunction*.

[19] Miedel A, Tegerstedt G, Maehle-Smidt M, Nyrén O, Hammarström M (2008). Symptoms and pelvic floor defects in specific compartments. *Obstetrics and Gynecology*.

[20] Ellerkmann RM, Cundiff GW, Melick CF, Nihira MA, Leffler K, Bent AE (2001). Correlation of symptoms with location and severity of pelvic organ prolapse. *American Journal of Obstetrics and Gynecology*.

[21] Takahashi T, Yamana T, Sahara R, Iwadare J (2006). Enterocele: what is the clinical implication?. *Diseases of the Colon and Rectum*.

[22] Chou Q, Weber AM, Piedmonte MR (2000). Clinical presentation of enterocele. *Obstetrics and Gynecology*.

[23] Mant J, Painter R, Vessey M (1997). Epidemiology of genital prolapse: observations from the oxford family planning association study. *British Journal of Obstetrics and Gynaecology*.

[24] South MMT, Stinnett SS, Sanders DB, Weidner AC (2009). Levator ani denervation and reinnervation 6 months after childbirth. *American Journal of Obstetrics and Gynecology*.

[25] Baumann P, Hammoud AO, McNeeley SG, DeRose E, Kudish B, Hendrix S (2007). Factors associated with anal sphincter laceration in 40,923 primiparous women. *International Urogynecology Journal and Pelvic Floor Dysfunction*.

[26] Klingele CJ, Bharucha AE, Fletcher JG, Gebhart JB, Riederer SG, Zinsmeister AR (2005). Pelvic organ prolapse in defecatory disorders. *Obstetrics and Gynecology*.

[27] Soligo M, Salvatore S, Emmanuel AV (2006). Patterns of constipation in urogynecology: clinical importance and pathophyiologic insights. *American Journal of Obstetrics and Gynecology*.

[28] Jelovsek JE, Barber MD, Paraiso MFR, Walters MD (2005). Functional bowel and anorectal disorders in patients with pelvic organ prolapse and incontinence. *American Journal of Obstetrics and Gynecology*.

[29] Shorvon PJ, McHugh S, Diamant NE, Somers S, Stevenson GW (1989). Defecography in normal volunteers: results and implications. *Gut*.

[30] Groenendijk AG, De Blok S, Birnie E, Bonsel GJ (2005). Interobserver agreement and intersystem comparison of the halfway system of baden and walker versus the pelvic organ prolapse-quantitation prolapse classification system in assessing the severity of pelvic organ prolapse. *Journal of Pelvic Medicine and Surgery*.

[31] Kelvin FM, Maglinte DDT, Hornback JA, Benson JT (1992). Pelvic prolapse: assessment with evacuation proctography (defecography). *Radiology*.

[32] Lukacz ES, Lawrence JM, Contreras R, Nager CW, Luber KM (2006). Parity, mode of delivery, and pelvic floor disorders. *Obstetrics and Gynecology*.

[33] Dietz HP (2008). The aetiology of prolapse. *International Urogynecology Journal and Pelvic Floor Dysfunction*.

